# Irreversible Plastic Flows and Sedimentary Ecological Entrapment: A Critical Review of Legacy Risks and Governance Strategies for Planetary Health

**DOI:** 10.3390/nano15201546

**Published:** 2025-10-10

**Authors:** Seong-Dae Moon, Su-Ok Hwang, Byeong-Hun Han, Dae-sik Hwang, Baik-Ho Kim

**Affiliations:** 1 NEB Co., Seoul 08504, Republic of Korea; neb.sdmoon@gmail.com (S.-D.M.); neb.dshwang@gmail.com (D.-s.H.); 2 Research Institute for Natural Sciences, Hanyang University, Seoul 04763, Republic of Korea; spring3974@naver.com; 3 Dongmoon ENT, Guro-gu, Seoul 08377, Republic of Korea; tttyy3@naver.com; 4 Department of Environmental Science, Hanyang University, Seoul 04763, Republic of Korea

**Keywords:** microplastics, nanoplastics, irreversible plastic transport, sedimentary entrapment, legacy pollutants, transdisciplinary governance, earth system risk assessment

## Abstract

Plastic pollution has emerged as a pervasive and systemic driver of ecological and biogeochemical disruption in freshwater and marine environments. Unlike natural materials that circulate within closed biogeochemical loops, synthetic polymers predominantly follow unidirectional and irreversible trajectories, a phenomenon we describe as “irreversible plastic transport.” These flows culminate in sedimentary entrapment, where plastics persist as long-term ecological stressors and potential vectors of contaminant transfer. Recent global syntheses indicate that sedimentary microplastic loads can exceed 27,000 particles/kg dry weight in certain river systems, highlighting the urgency of sediment-inclusive risk assessments. This review synthesizes interdisciplinary findings to conceptualize plastics as both pollutants and governance challenges. We highlighted the dominant transport pathways of micro- and nanoplastics and emphasize that sedimentary sinks are critical long-term retention zones. Current monitoring frameworks often underestimate sedimentary burdens by focusing on surface water and overlooking subsurface ecological legacies. We propose an integrated governance approach combining cross-media monitoring, Earth system modeling, and adaptive policies to address these persistent synthetic agents. Embedding plastic dynamics within comprehensive risk assessment frameworks is essential for sustainable water management during the Anthropocene. Our synthesis supports risk-based decision-making and encourages proactive, transdisciplinary global governance strategies that integrate sediment-focused monitoring and long-term ecological risk management.

## 1. Introduction

Plastic pollution has evolved from a marine-centric concern to a planetary-scale environmental crisis affecting terrestrial, freshwater, and marine environments. Although oceans have historically been regarded as the final sinks for plastic debris, recent research has highlighted the critical yet underappreciated role of rivers, not only as conduits but also as active retention zones for plastic materials [1,2]. These dual roles complicate the efforts to trace, monitor, and mitigate plastic flows across interconnected ecosystems.

Since the onset of mass production in the 1950s, more than 10 billion metric tons of synthetic polymers have been manufactured, including plastics in virtually every industrial sector from packaging and textiles to medicine and construction [3,4,5,6,7]. Their high durability, low cost, and lightweight nature have facilitated their widespread adoption; however, these properties have also entrenched a linear, non-recoverable (irreversible) flow of materials that diverges from natural recycling systems. Unlike biogeochemically integrated cycles, such as carbon or nitrogen, plastics predominantly follow a one-way trajectory [8,9,10].

Traditionally, rivers and freshwater systems have been viewed as passive conduits transporting plastic waste from terrestrial landscapes to the marine environment. However, recent empirical evidence has reframed this understanding, showing that rivers also function as active ecological traps where microplastics are retained via sedimentation, sorption onto organic particles, biofilm incorporation, and hydrodynamic deceleration [1,11,12]. Physical structures such as weirs and dams, along with seasonal flow variation and biotic interactions, further enhance the in-stream retention capacity [13,14,15]. Accordingly, rivers must be understood not merely as linear transport systems, but as dual-function interfaces that both convey and entrap plastics, ultimately modulating the flux of synthetic pollutants to coastal and marine ecosystems.

In this review, we use the term “irreversible plastic transport” to describe the predominantly linear and non-recoverable movement of plastic materials through the environment. Unlike biogeochemical cycles, such as carbon or nitrogen, which involve feedback loops and ecosystem-level recycling, plastic flows lack intrinsic biological or chemical return pathways. This concept is fundamental to understanding plastics as legacy pollutants during the Anthropocene.

**Figure 1:**
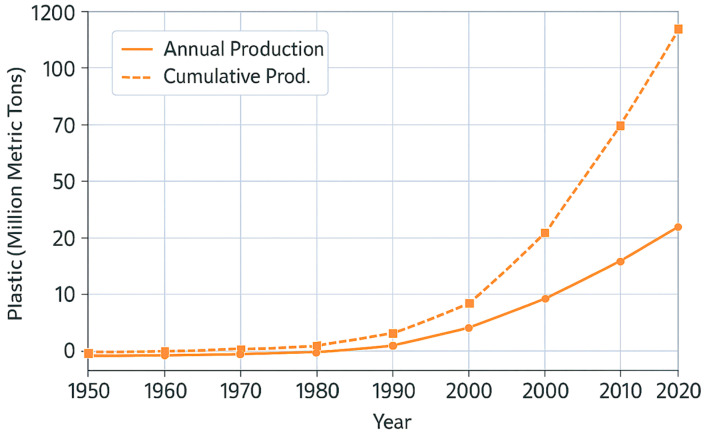
Global trends in plastic production and cumulative accumulation from 1950 to 2020. This figure presents harmonized time-series data compiled from Our World in Data, GRID-Arendal, OECD, and Green Post Korea [25,26,27,28], illustrating the exponential rise in annual plastic production and cumulative plastic stocks. The data underscore the global shift toward a plastic-dependent society and highlight the persistence of plastics as a defining hallmark of the Anthropocene.

As illustrated in Figure 1, this irreversible transport pathway has led to the extensive accumulation of plastic waste in soils, riverbeds, estuaries, oceans, and the atmosphere [16,17,18]. The molecular properties of plastics, including high hydrophobicity, low reactivity, and resistance to photolytic and microbial degradation, enable them to persist for decades or even centuries [19,20,21]. Plastics accumulate in sediments and organisms, traverse trophic levels, and have been detected in remote environments such as Arctic snow, deep-sea trenches, and human tissues [11,22,23,24]. Importantly, this review highlights nanoplastic properties and detection challenges, directly linking environmental dynamics with the broader field of nanomaterials science. By situating sedimentary plastics within the analytical and conceptual frameworks of nanomaterials, this study underscores the journal’s focus on nanoscale materials and their ecological significance.

Plastics act as vectors for environmental contaminants by adsorbing heavy metals and persistent organic pollutants. This composite behavior amplifies the ecotoxicological footprint, disrupting microbial communities, nutrient cycling, and food web dynamics [29,30,31,32]. Consequently, researchers have increasingly conceptualized plastics not merely as pollutants but also as agents within a novel synthetic material cycle—an anthropogenic system disconnected from natural feedback mechanisms and characterized by long-term ecological and geochemical legacies [11,20,33].

This review aims to elucidate the mechanisms and consequences of ecological entrapment of plastics, with a particular emphasis on long-term retention of microplastics and nanoplastics in freshwater and sedimentary systems. As a descriptive and integrative synthesis, this review does not present new statistical analyses or generate original datasets. Instead, it synthesizes findings from over 180 peer-reviewed studies published between 2000 and 2024 to build a transdisciplinary understanding of plastic transport, sediment entrapment, and governance challenges during the Anthropocene. This non-empirical approach is particularly suited for analyzing the cumulative system-level effects of irreversible plastic transport and its long-lasting ecological legacies across aquatic compartments.

To guide this synthesis, we address the following key research questions:

How do plastics move through freshwater and marine systems as irreversible flows, and what mechanisms drive their transport?

How do riverine and sedimentary systems function as ecological entrapment zones, and what are their implications for long-term ecosystem health?

What are the potential legacy effects of sedimentary plastics, and how do they contribute to cumulative ecological risks?

Which governance and monitoring strategies effectively address the systemic and transboundary nature of plastic pollution?

To address these questions, we adopted a narrative synthesis methodology that integrates insights from environmental science, materials research, regulatory studies, and environmental ethics into a coherent analytical framework. We explore the dominant pathways by which plastics are transported through freshwater and marine systems, and examine how sediments, particularly those in rivers, act as ecological entrapment zones, facilitating long-term retention and potential re-exposure. We also analyzed the long-term implications of legacy plastics, focusing on micro- and nanoplastics, on aquatic environments and ecosystem health. Finally, we discuss the interdisciplinary strategies required to address the global plastic crisis, emphasizing the importance of standardized protocols, advanced modeling approaches, and comprehensive policy reforms.

Relevant literature was identified using structured keyword searches in Web of Science and Scopus, employing terms such as plastic pollution, river entrapment, microplastics, and sediment retention. Only empirical and synthesis-based studies addressing transport mechanisms, sedimentation dynamics, degradation pathways, and ecological impacts across freshwater and marine systems were included. Editorials, opinion articles, preprints, and non-peer-reviewed sources were excluded to ensure scientific reliability.

## 2. Methods

### 2.1. Literature Search Strategy

This review employs a narrative synthesis approach to integrate peer-reviewed literature addressing the environmental dynamics, transport pathways, sediment retention, and governance frameworks of micro- and nanoplastics. Systematic searches were conducted using Web of Science, Scopus, and Google Scholar with the following keywords: microplastic, nanoplastic, transport, sediment retention, ecological entrapment, irreversible plastic transport, legacy pollution, plastic governance, and Anthropocene. Boolean operators and advanced filters were applied to refine the search outputs. The search focused on English-language publications from 2000 to 2024, with emphasis on studies published after 2018, to capture the most recent scientific and methodological advances.

### 2.2. Inclusion and Exclusion Criteria

We included peer-reviewed journal articles, comprehensive review papers, and institutional reports (e.g., United Nations Environment Programme (UNEP), Organization for Economic Co-operation and Development (OECD), and World Bank) that addressed plastic pollution from physical, ecological, or governance perspectives. Literature focusing on transport mechanisms, sedimentary retention dynamics, degradation pathways, ecological impacts, and policy interventions was prioritized. Editorials, preprints, conference abstracts, non-peer-reviewed materials, and opinion pieces were excluded to maintain scientific rigor. From an initial pool of over 180 publications, approximately 120 were selected based on their relevance to the four guiding research themes outlined in Section 1.

### 2.3. Analytical Scope and Indicator Synthesis

As a descriptive narrative review, this study did not involve formal statistical testing or generation of new primary datasets. Instead, we synthesized conceptual frameworks and key ecological indicators discussed in the reviewed literature. These indicators include sediment burial rates (reflecting long-term retention), biofilm-mediated stabilization, hydrodynamic retention thresholds (linked to fluvial velocity and geomorphic features), and sorption-based contaminant loading (demonstrating plastics as chemical vectors). Additionally, we examined how the polymer type, particle size, and surface properties influenced the fate and transport across interconnected environmental compartments. Together, these indicators provide an integrated perspective of plastic persistence and ecological entrapment.

### 2.4. Visualization and Data Sourcing

Figures in this review are categorized into two main types: empirical data visualizations and conceptual schematics. Figure 1 presents harmonized time-series data compiled from authoritative sources, including Our World in Data, GRID-Arendal, OECD Global Plastics Outlook, and Green Post Korea [25,26,27,28]. Where minor data gaps existed, linear interpolation was applied using Microsoft Excel to maintain temporal continuity and data coherence.

**Figure 2:**
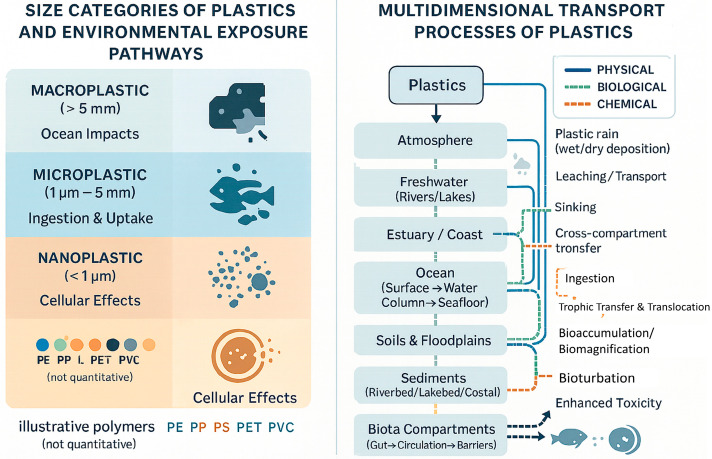
Integrated classification and transport mechanisms of plastics across environmental compartments.

**Figure 3:**
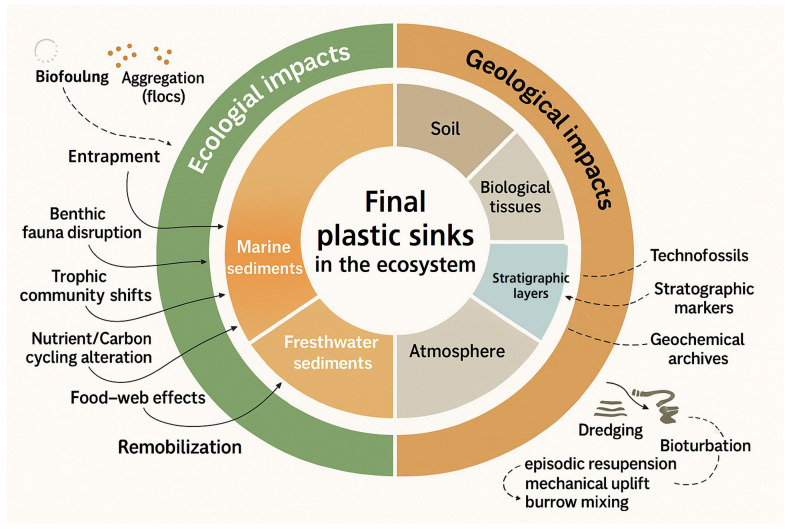
Ecological entrapment and sedimentary retention of plastics. This schematic depicts how plastics become entrapped in sediments via biofouling, aggregation, and burial, and how they may subsequently be remobilized by hydrodynamic disturbances, dredging operations, or bioturbation. The figure integrates findings from [38,89,91].

**Figure 4:**
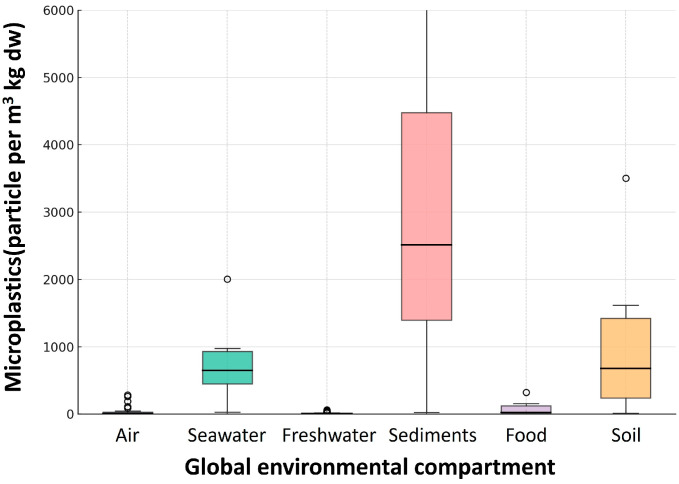
Distribution of microplastic particle concentrations across environmental compartments. Box plots synthesized from peer-reviewed studies (detailed in Supplementary Table S2), illustrating the variability and distribution of concentrations (particles per m^3^ or kg dw) in air (n = 31), seawater (n = 41), freshwater (n = 6), sediments (n = 12), food (n = 5), and soil (n = 6). The plots highlight higher concentrations and wider variation in sediments and soils compared to narrower distributions in air and seawater. Open circles denote study-level outliers (values beyond the whiskers), including reported maxima. This figure represents an integrative data synthesis and is not a formal meta-analysis.

**Figure 5:**
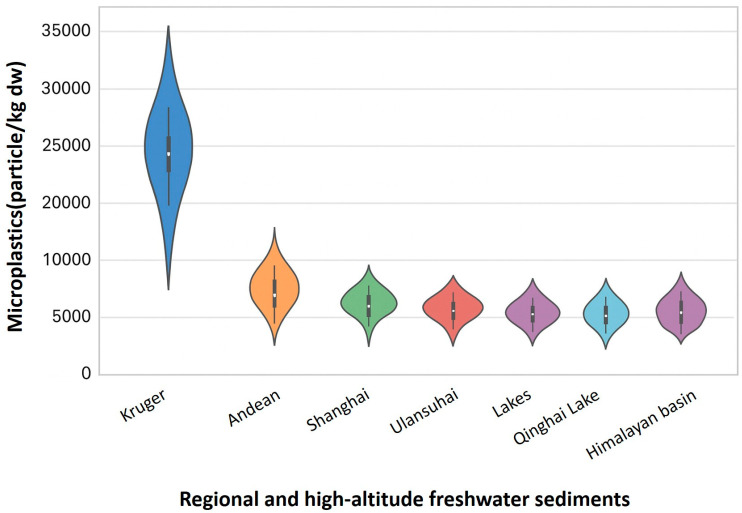
Distribution of microplastic concentrations in regional and high-altitude freshwater sediments. Violin plots synthesized from 7 peer-reviewed studies (2017–2025), with source data detailed in Supplementary Table S3. This figure represents an integrative data synthesis rather than a formal statistical meta-analysis.

**Figure 6:**
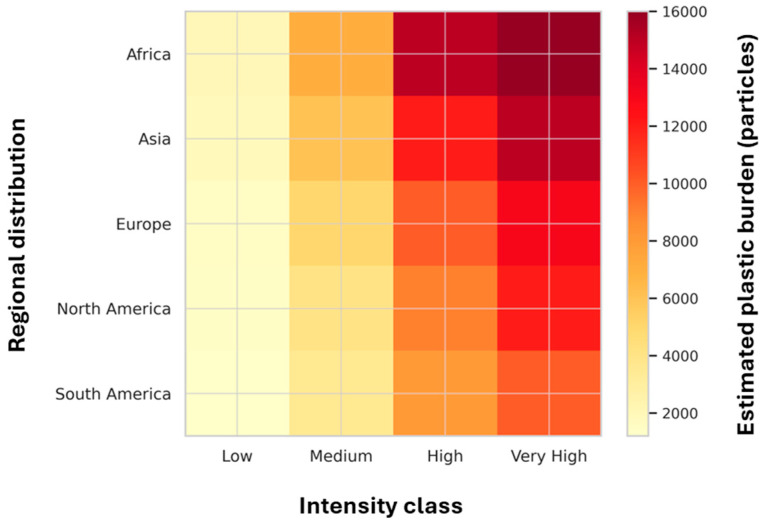
Regional heatmap showing estimated plastic burdens across major global regions. Color gradients represent approximate particle concentrations (particles per m^3^ or kg dw), categorized by conceptual intensity classes: Low (<100 particles/kg dw or m^3^), Medium (100–1000), High (1000–10,000), and Very High (>10,000). This figure represents an integrative data synthesis from the reviewed literature and is not a formal meta-analysis.

**Figure 7:**
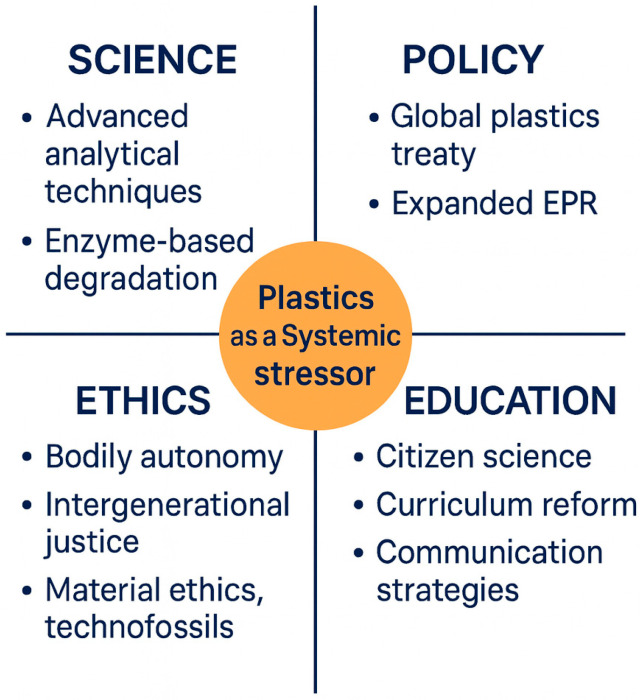
Integrated governance framework for systemic plastic pollution management. This transdisciplinary model highlights science, policy, ethics, and education as intersecting domains that must be integrated to manage plastics as systemic stressors. The framework is informed by Earth system governance theory and sustainability science. EPR = Extended Producer Responsibility.

Figures 2–7 consist of original conceptual diagrams and integrated empirical visualizations that synthesize systemic, ecological, and governance perspectives derived from the reviewed literature. Specifically, Figure 2 provides a consolidated schematic that integrates plastic size classification with multidimensional transport mechanisms, combining particle-scale attributes with physical, biological, and chemical pathways across environmental compartments. Figure 3 illustrates ecological entrapment and sedimentary retention dynamics, while Figures 4 and 5 show cross-compartmental distributions through a box plot and a violin plot, respectively. Figure 6 presents regional burden heat maps, and Figure 7 highlights a systems-level governance framework.

All visualizations were developed using Adobe Illustrator 2023 and BioRender (BioRender.com; academic publication license; accessed on 18 August 2025), with supplementary data processing conducted in Microsoft Excel, where applicable. This consolidated visualization strategy reduces redundancy, enhances conceptual integration, and improves consistency across figures, thereby facilitating clearer interpretation of systemic plastic flows.

This consolidated schematic combines size-based classification (macro-, micro-, and nanoplastics) with major transport pathways. The left panel categorizes plastics by particle size and environmental behavior, including suspension, sedimentation, ingestion, and cellular uptake. The right panel depicts multidimensional transport processes, including physical (runoff, sedimentation, atmospheric deposition), biological (ingestion, trophic transfer, fecal pellet transport), and chemical (photodegradation, microbial transformation) pathways. Together, these panels illustrate how particle properties and environmental forces jointly determine cross-compartmental plastic dynamics.

This methodological framework, which encompasses systematic literature search, critical selection, indicator synthesis, and integrative visualization, ensures analytical coherence and scientific transparency. It also facilitates interdisciplinary interpretation of the transport, retention, and governance of micro- and nanoplastics across environmental and policy domains.

## 3. Plastic Types and Physicochemical Properties

This section addresses the following question: What material properties govern environmental persistence of microplastics? To answer this question, we explored the size-based classification, particle morphology, surface chemistry, and degradation behavior. In this literature-based review, this section synthesizes published findings to explain environmental persistence and mobility of plastic materials.

Understanding plastic transport and persistence requires examination of fundamental physicochemical traits. Key properties such as density, hydrophobicity, crystallinity, and surface reactivity determine whether plastics float, sink, adsorb contaminants, or degrade over time [4,5,34,35,36].

Plastics are not a homogeneous material group, but comprise diverse polymer types with distinct structural and chemical characteristics. Common plastics include polyethylene (PE), polypropylene (PP), polystyrene (PS), polyethylene terephthalate (PET), and polyvinyl chloride (PVC), each with unique implications for environmental mobility and persistence [37,38,39]. For instance, low-density polymers such as PE and PP often remain buoyant, facilitating widespread aquatic dispersal, whereas denser polymers such as PET and PVC tend to sink, leading to sedimentary accumulation.

Plastics are also classified by particle size and form, which critically influence their environmental behavior and ecological impact. This review focuses on microplastics (<5 mm) and nanoplastics (<100 nm), which pose distinct challenges due to their persistence and bioavailability. Microplastics originate either as primary microplastics (e.g., microbeads and industrial abrasives) or secondary microplastics from the fragmentation of larger debris via photodegradation, mechanical abrasion, and weathering [8,40,41]. Although macroplastics (>5 mm) have been acknowledged as precursors, they were not analytically or conceptually examined in this study.

Microplastics have been widely detected in virtually all environmental compartments, including freshwater bodies, sediments, soils, air, and human tissues [42,43,44]. Of particular concern are nanoplastics, which can be derived from further fragmentation of microplastics or can be directly engineered. Due to their nanoscale dimensions, nanoplastics can cross cellular membranes, evade conventional filtration, and potentially disrupt intracellular metabolic processes [45,46,47,48,49,50,51,52].

Despite their ecological significance, nanoplastics remain poorly characterized in most field studies owing to analytical limitations and a lack of standardized detection protocols [40,53,54,55,56,57,58]. Advancing this field requires improved sampling methodologies, advanced instrumentation, and mechanistic studies targeting ultrafine particles as emerging contaminants.

Environmental persistence of plastics is largely linked to their resistance to photolytic, microbial, and oxidative degradation [21,57,58,59,60,61,62]. Moreover, their surface properties enhance their role as contaminant vectors: many plastics adsorb persistent organic pollutants, heavy metals, and antibiotics, which can bioaccumulate and magnify ecological risks [30,63,64,65,66,67,68,69,70,71,72]. Additives such as plasticizers and flame retardants further contribute to their toxicological complexity through leaching [5,36,73,74,75,76].

As summarized in Table 1, the physicochemical traits (durability, hydrophobicity, density, chemical additives, and surface area) determine fate and impact of each polymer on environmental systems. Figure 2 complements this overview by visually illustrating the influence of these properties on plastic transport, fate, and ecological exposure. Collectively, these characteristics, reinforced by recent reviews (e.g., ref. [57,58,70]), shape the long-term behavior of plastics, underpinning non-cyclic plastic flows and legacy effects in ecosystems. Understanding these characteristics is critical for developing effective monitoring, risk assessment, and governance strategies.

**Table 1:** Physicochemical properties of plastics driving environmental persistence and ecological risks.

Physicochemical Property	Scientific Description	Ecological Function and Implications	References
Durability	High molecular stability; resists thermal, photolytic, and microbial degradation.	Enables long-term persistence and accumulation in soils, sediments, and aquatic environments.	[21,57,58,62,77,78]
Hydrophobicity	Non-polar nature facilitates sorption of hydrophobic organic pollutants and limits water solubility.	Enhances environmental mobility and interaction with co-contaminants, promoting bioaccumulation.	[67,68,69,79,80,81]
Density and buoyancy	Density determines floating or sinking behavior in aquatic systems, affecting vertical distribution.	Influences exposure risk for pelagic versus benthic organisms; contributes to sediment entrapment.	[40,42,57,82,83,84]
Chemical additives	Includes plasticizers, flame retardants, and endocrine disruptors such as BPA and PFAS.	Leached chemicals cause endocrine disruption, reproductive toxicity, and bioaccumulation in wildlife.	[5,23,34,62,75,76]
Adsorptive capacity	Large surface area and surface chemistry enable adsorption of POPs, antibiotics, and metals.	Facilitates pollutant transfer across trophic levels; increases ecological toxicity and bioavailability.	[30,63,67,70,71,72,85]
Surface area-to-volume ratio	Fragmentation increases surface area, enhancing reactivity and cellular interactions.	Promotes oxidative stress, inflammatory responses, and cellular disruption in exposed biota.	[44,46,50,51,52,53,86]

## 4. Transport Pathways of Plastics Across Freshwater and Marine Ecosystems

Building on the material properties described in the previous section, this section explores ecological transport pathways of plastics in terrestrial, freshwater, estuarine, and marine environments. These pathways are shaped not only by the physicochemical properties of plastics but also by hydrological forces, geomorphological settings, and anthropogenic inputs. Recent studies have highlighted roles of atmospheric deposition, biological transfer, and chemical transformation in driving cross-boundary plastic dispersal, emphasizing complex intercompartmental dynamics and enhanced ecological risks [38,57,58,62,74,87]. Understanding how plastics move through these compartments is essential for identifying points of accumulation, persistence, and ecological risks.

Plastics typically enter the environment through multiple sources, including urban runoff, wastewater discharge, industrial leakage, mismanaged waste, and atmospheric deposition [8,16,43]. Once released, their movement is governed by surface flow dynamics, buoyancy, and interactions with sediments and biota. For example, low-density polymers, such as PE and PP, often remain suspended in water columns or float, allowing for rapid downstream or oceanic dispersal. In contrast, denser plastics, such as PVC and PET, are prone to settling in riverbeds and coastal sediments, becoming part of long-term depositional environments [11,12,88].

Freshwater systems, particularly rivers and lakes, act as conveyors and sinks for microplastics. Historically regarded as passive transport media, rivers are increasingly being recognized as dynamic filters that trap substantial quantities of microplastic particles. Obstructions, such as dams, weirs, riparian vegetation, and sediment bars, can significantly enhance the retention of these particles within the river network [1,36]. Seasonal fluctuations in flow regimes also affect transport efficiency and re-mobilization. During high-flow events, microplastics can be flushed downstream or deposited in floodplains, whereas during low-flow periods, they tend to accumulate in sediments along channel margins [89,90].

Estuarine and coastal zones are complex transition areas where freshwater and marine plastic pathways converge. In brackish environments, plastics may undergo physical and chemical transformations such as salinity-induced aggregation, biofouling, or fragmentation, which alter their transport behavior and bioavailability [67,68,69,70,71,72,82,91]. Tidal pumping, stratification, and estuarine circulation patterns contribute to the bidirectional movement and entrapment of plastics, often resulting in their long-term accumulation in sediments [16,17].

Atmospheric transport of plastics, particularly micro- and nanoplastics, has recently emerged as a significant pathway. Wind erosion of terrestrial surfaces, aerosolization from wastewater effluent, and urban dust emissions can mobilize plastic particles into the air, allowing them to be transported over long distances and deposited into remote ecosystems—including high-altitude lakes, polar ice, and marine surfaces [43,92,93,94]. This vertical flux challenges the traditional assumption that plastics are primarily waterborne.

The integrated Figure 2 illustrates major pathways through which plastics move across environmental compartments, linking size-based classifications with physical, biological, and chemical processes such as suspension, sedimentation, aggregation, ingestion, trophic transfer, and photochemical transformation. Critical ecological interfaces, including riparian zones, estuaries, and atmospheric exchange, are highlighted as key nodes of retention and transfer. Table 2 complements this figure by systematically categorizing the transport modalities, associated mechanisms, and ecological impacts, thereby reinforcing the multifaceted and cross-boundary nature of plastic dispersal.

In terrestrial and aquatic food webs, microplastics are taken up by organisms and pass through trophic levels through ingestion and excretion. This biological movement facilitates their transfer across systems, from sediments to surface water, and even into airborne pathways via fecal pellets and volatilization [22,58,62,84,91,95,96].

Recent global assessments (Supplementary Table S1) have revealed striking variability in microplastic concentrations across aquatic compartments. River water levels can reach up to 4000 particles per liter (PC/L), whereas sedimentary loads have been documented as high as 27,259 particles per kilogram dry weight (PC/kg dw) in heavily impacted systems [97,98]. These findings emphasize the necessity for comprehensive transport pathway analyses that integrate water column dynamics and sedimentary retention across connected freshwater and marine systems.

**Table 2:** Cross-media transport mechanisms and ecological impacts of plastics.

Transport Modality	Environmental Medium	Mechanisms and Examples	Ecological Impacts	References
Physical transport	Atmosphere, hydrosphere, lithosphere	Wind uplift; rainfall and surface runoff; ocean currents; cryospheric drift	Cross-boundary dispersal; long-range transport; deposition in remote and pristine regions	[43,57,58,62,99,100]
Biological transport	Aquatic and terrestrial biota	Ingestion; trophic transfer; fecal pellet transport; placental translocation	Bioaccumulation; physiological stress; trophic web disruption	[22,58,62,84,91,96]
Chemical transformation	UV exposure, oxidative, microbial environments	Photodegradation; thermo-oxidation; enzymatic depolymerization (e.g., PETase)	Generation of micro- and nanoplastics; enhanced pollutant sorption and bioavailability	[67,68,69,70,71,72,101,102,103]

To provide comprehensive overview, Table 2 classifies three major transport modalities—physical, biological, and chemical—along with representative mechanisms (e.g., wind uplift, UV degradation, and trophic transfer), their associated environmental media, and corresponding ecosystem-level impacts. This tabular synthesis supports systems-level understanding of cross-boundary plastic dynamics and ecological exposure pathways.

## 5. Sediments as Ecological Entrapment Zones for Plastics

This section presents qualitative synthesis of key insights drawn from peer-reviewed studies and policy reports organized around the four guiding questions introduced in the Introduction. This review is descriptive in nature and does not involve statistical testing; instead, it interprets published findings through integrative reasoning. No original statistical analysis was conducted; rather, findings were interpreted through conceptual integration and comparative reasoning across multiple knowledge domains (e.g., sediment science, hydrology, and environmental governance). The figures illustrate representative patterns, frameworks, and mechanisms identified in the literature.

Based on transport dynamics described in the previous section, this section focuses on ecological entrapment and retention of plastics in sedimentary environments. As plastics move through terrestrial, freshwater, estuarine, and coastal systems, many are intercepted by ecological filters and physical barriers, ultimately leading to their long-term deposition in benthic zones. Sedimentary retention has significant implications for ecosystem health, biogeochemical cycles, and the emergence of plastic legacy effects [38,104,105].

Sediments function as critical long-term sinks for microplastics and their fragmented derivatives, facilitating ecological entrapment primarily through physical mechanisms. Once deposited, microplastics may become buried by successive sedimentation or stabilized via microbial biofilms, riparian vegetation, and hydrodynamic forces [17,89]. In fluvial systems, this retention is further governed by geomorphological features, such as meanders, oxbows, floodplains, and impoundments, which reduce the flow velocity and enhance the deposition probability [36,88]. Recent research has demonstrated that substantial fractions of microplastics can persist within freshwater sediments for prolonged periods, undergoing remobilization primarily during extreme hydrological disturbances [106,107,108].

Estuarine and coastal sediments act as convergence zones for freshwater-transported and marine plastic. Biofouling and salinity-induced aggregation increase the density of floating plastics, causing them to settle into soft sediments, where they become partially or fully buried [82,91,109]. These entrapment zones are often poorly monitored and represent long-term reservoirs of plastic debris, including fibers and fragments, which can persist for decades [35,38,110].

Recent global syntheses have demonstrated substantial variability in microplastic loads within riverine and freshwater sediments. For example, sediment concentrations have been reported to be as high as 27,259 PC/kg dw in the Kruger National Park rivers in South Africa [97], 6383 PC/kg dw in the Andean rivers in Peru [98], and 4250 PC/kg dw in Shanghai rivers [111]. Similarly, lake sediments in the Peruvian Andes and China’s Lake Ulansuhai showed concentrations up to 3680 and 3767 ± 1626 PC/kg dw, respectively [112,113]. These figures, drawn from a recent global dataset (Supplementary Table S1), highlight the urgent need to consider sediments as critical ecological entrapment zones. We expanded Supplementary Table S1 to include additional data from 2015–2025, covering marine, freshwater, atmospheric, food, and soil compartments, thereby strengthening the global perspective on microplastic concentrations.

Sediments containing legacy plastics also act as potential sources of re-exposure, particularly when disturbed by storms, dredging, or bioturbation. Understanding physical retention and remobilization dynamics of these materials is crucial for comprehending their full environmental persistence and long-term ecological risks. As illustrated in Figure 3, ecological entrapment and sedimentary retention involve processes such as biof I confirmouling, aggregation, and burial, while hydrodynamic disturbances, dredging operations, or bioturbation can trigger episodic resuspension (see [38,89,91]). These processes underscore that sediments function not merely as passive reservoirs, but also as dynamic zones actively modulating plastic fate.

Table 2 categorizes principal transport vectors—physical, biological, and chemical—governing plastic movement across atmospheric, aquatic, and terrestrial systems. Each vector is linked to representative mechanisms (e.g., wind uplift, trophic transfer, and UV degradation) and their ecological implications, including food web disruption and the generation of secondary microplastics. This integrated classification framework provides robust foundation for understanding cross-boundary plastic dynamics and underscores connectivity among diverse ecosystems.

Table 3 summarizes current understanding of how plastics persist across environmental sinks, including the atmosphere, marine sediments, agricultural soils, and biological tissues, and outlines their long-term ecological consequences. This expanded system-level classification, reinforced by recent integrative reviews [57,58,62], strengthens concepts of plastic legacy and ecological entrapment. This further highlights cumulative risks and potential feedback effects within and across ecosystems, emphasizing their enduring impacts on biogeochemical cycles, biodiversity, and human health.

**Table 3:** Environmental sinks of plastics: persistence pathways and system-level impacts.

Final Sink	Entry Pathways	Persistence Mechanisms	System-Level Impacts	References
Atmosphere	Wind transport, urban emissions, synthetic textiles shedding	Global dispersal, ice trapping, dry and wet deposition	Respiratory health risks, contamination of remote and pristine areas	[43,58,62,99,114]
Marine sediments	Sinking via biofouling, marine snow, particle aggregation	Anaerobic conditions, deep burial, long-term entrapment	Disruption of benthic fauna, alteration of carbon and nutrient cycling	[4,41,57,67,68,82]
Agricultural soils	Plastic mulch application, sludge amendment, irrigation runoff	Soil embedding, bioturbation, slow abiotic and microbial degradation	Soil fertility decline, microbial community shifts, crop uptake and food chain entry	[35,58,84,115,116]
Biological tissues	Ingestion and inhalation via air, water, and food	Cellular uptake, translocation across organs, systemic circulation	Hormonal disruptions, inflammatory responses, intergenerational health effects	[22,50,51,52,86,96]
Stratigraphic layers	Sediment burial, fossilization processes	Geological preservation, technofossil formation	Long-term environmental archives, markers of the Anthropocene epoch	[4,70,72,117,118]

To strengthen global perspective of sedimentary and aquatic microplastic retention, we compiled concentration data from 29 key peer-reviewed studies and reviews published between 2017 and 2025 (Supplementary Table S1). This integrated synthesis revealed substantial variability in microplastic contamination levels across different environmental compartments, emphasizing critical need for sediment-inclusive monitoring strategies, as discussed in subsequent sections.

To further illustrate spatial and environmental variability, we provide box plot summary (Figure 4) that synthesizes sediment concentration data from diverse global sites, including regional and high-altitude freshwater sediments.

## 6. Ecological Legacies of Sedimentary Plastics

Plastics entrapped in sediments represent one of the most persistent and under-recognized dimensions of global plastic pollution. Once deposited, these materials interact dynamically with benthic ecosystems, become integrated into biological processes, and alter sedimentary biogeochemistry. Over time, sediments evolve into latent reservoirs of plastics and associated contaminants capable of releasing pollutants through natural or anthropogenic disturbances. This section synthesizes current understanding of ecological feedback and legacy impacts of sedimentary plastic entrapment and highlights critical gaps in monitoring frameworks that must be addressed to capture the true extent of plastic pollution.

### 6.1. Biological and Biogeochemical Feedbacks

Building on the sedimentary entrapment mechanisms outlined in Section 5, this subsection focuses on long-term ecological consequences of plastic retention in benthic environments. Once deposited, micro- and nanoplastics are not passively buried; rather, they become actively integrated into dynamic biological and biogeochemical processes that shape sediment function and ecosystem health. In situ observations have revealed that deposit feeders, benthic invertebrates, and microbial mats incorporate microplastics into burrows or bio-aggregates, facilitating spatial redistribution and enhancing fragmentation [119,120,121]. These interactions extend the residence time of plastics within sediments and promote trophic transfer of associated contaminants [63,122,123].

Recent global data support these findings, with sedimentary microplastic concentrations reaching extreme levels in several freshwater and lacustrine systems. For example, lake sediments in Lake Ulansuhai (China) contained up to approximately 3767 ± 1626 PC/kg dw [112], and sediments in Peruvian Andean lakes showed concentrations up to 3680 PC/kg dw [113]. Riverine sediments in urban and semi-natural systems, such as Kruger National Park rivers (South Africa) and Shanghai rivers (China), have been reported at 27,259 and 4250 PC/kg dw, respectively [97,111]. These elevated levels illustrate potential of plastics to be bioavailable to benthic communities for extended periods and to mediate long-term ecological feedback.

Moreover, presence of entrapped plastics has been shown to alter local sediment biogeochemistry by modifying oxygen gradients, reshaping microbial community composition, and affecting contaminant fluxes [29,124]. Such alterations can exacerbate nutrient imbalances and enhance persistence of co-contaminants, reinforcing the role of plastics as vectors of legacy stressors in sedimentary systems. Beyond ecological consequences, sedimentary plastics can adversely affect human systems by contaminating fisheries, reducing agricultural soil fertility and crop safety, and posing risks to potable water supplies when sediments are remobilized. These pathways highlight sedimentary plastics as both ecological and public health stressors.

A cross-compartmental violin plot (Figure 5) further visualizes concentration distributions of microplastics across various environmental media, including air, seawater, freshwater, sediments, food, and soil, underscoring compartment-specific variability and differential exposure risks.

### 6.2. Legacy Effects and Re-Exposure Risks

Despite increasing awareness, long-term legacy effects of plastic entrapment in sediments remain poorly understood. Observational studies have highlighted fundamental mismatch between surface water monitoring, where most plastic assessments are concentrated, and the actual environmental burden of subsurface sediments [89,106]. Sedimentary compartments act as latent reservoirs capable of releasing plastic during flood events, dredging, or bioturbation, thereby prolonging ecological and human exposure [107].

Global synthesis of sedimentary microplastic concentrations (Supplementary Table S1) underscores this concern, with recorded values varying from as low as approximately 25 PC/kg dw in some riverine sediments to over 27,000 PC/kg dw in heavily impacted systems. These disparities illustrate not only spatial variability, but also potential for high-risk “hotspots” of legacy pollution that remain largely unaddressed in current risk assessments.

Field-based research further indicates that sediment entrapment amplifies challenge of estimating cumulative impacts, especially when plastics sorb environmental contaminants, such as heavy metals or persistent organic pollutants [63,86,124]. Moreover, fine-grained plastic fractions and microhabitats with high plastic loads are often underrepresented in standard monitoring protocols, leading to a systematic underestimation of exposure and risk.

To address these gaps, it is imperative to implement sediment-inclusive stratified monitoring strategies that integrate both spatial and vertical sediment layers [5,125]. By embedding benthic compartments into national and international surveillance frameworks, policymakers and researchers can more accurately assess long-term ecological legacy of plastic pollution and prioritize remediation efforts.

## 7. Monitoring Frameworks and Methodological Gaps

Despite synthesis of more than 120 peer-reviewed studies, several key limitations and knowledge gaps have hindered formulation of effective interdisciplinary responses to plastic pollution. These limitations span empirical, methodological, and socio-political domains, each of which constrains policy innovation and scientific understanding.

### 7.1. Empirical Gaps in Field-Based and Long-Term Observations

A critical shortfall is the limited availability of long-term field-based data on micro- and nano-plastics, particularly for freshwater and sedimentary systems. Nanoplastics remain especially under-monitored owing to analytical constraints [53,63], although recent advances in detection techniques such as advanced spectroscopy, thermal analysis, and nanoparticle tracking are beginning to close this gap [54]. Nonetheless, few studies have quantitatively documented nanoplastic retention, transformation, and ecological effects over time. Furthermore, sediment-specific degradation models remain underdeveloped and often exclude key biological processes such as microbial mediation and bioturbation [36,103,126].

Recent global data syntheses have illustrated stark discrepancies in sedimentary plastic burdens across regions. Concentrations in freshwater river sediments range from as low as 25 PC/kg dw to over 27,000 PC/kg dw, depending on urbanization, hydrodynamic regimes, and land-use pressures ([97,98], Supplementary Table S1). Such wide variability highlights the necessity for standardized, long-term monitoring programs that include sediment cores and vertical stratification rather than focusing solely on surface water layers.

Field observations [89,121,127] have demonstrated how microplastic fragments accumulate in sediments and are incorporated into benthic invertebrate burrows [91], thereby reinforcing reality of ecological entrapment. However, these studies remain isolated, emphasizing urgent need for harmonized, multi-site, and long-term monitoring campaigns that account for spatial heterogeneity and sedimentary depth profiles.

### 7.2. Methodological Limitations in Sediment Monitoring

Current monitoring strategies focus heavily on surface waters, neglecting sedimentary, atmospheric, and biological compartments. This systematic bias leads to a severe underestimation of the total plastic burden in aquatic environments [5,127]. Analytical inconsistencies ranging from particle size classification to detection methods further hinder cross-study comparisons and large-scale syntheses [82,128].

Global variability in sediment concentrations, such as 4250 PC/kg dw reported in Shanghai River sediments [111] or 3680 PC/kg dw in Peruvian Andean lakes [113], underscores the limitations of the current surveillance frameworks. Without sediment-inclusive monitoring, critical “hot spots” of legacy pollution remain undetected, thereby compromising effective policy design and intervention planning.

Promising regional initiatives have demonstrated feasibility of sediment-inclusive monitoring strategies. For example, the European Union’s Marine Strategy Framework Directive (MSFD) mandates sediment monitoring of microplastics as part of its comprehensive marine litter surveillance framework [125]. Similarly, the Thames River Programme in the United Kingdom integrates sediment sampling with long-term assessments, providing valuable empirical baselines [89]. Scaling such models to national and transboundary river systems can significantly improve our understanding of legacy plastic effects and inform targeted mitigation efforts. Notably, regional initiatives such as the Thames River sediment monitoring program in the United Kingdom, the Han River Basin Management Plan in South Korea, and the European Union’s MSFD demonstrate the practical feasibility and effectiveness of integrated multi-compartment surveillance approaches. These cases illustrate how local governance adaptations can serve as scalable models for global application.

To strengthen this global perspective, a regional heatmap (Figure 6) illustrates the estimated plastic burdens across major geographic regions, highlighting the key monitoring gaps and potential hotspots of legacy plastic accumulation.

### 7.3. Regional Governance Disparities and Capacity Gaps

Regions such as Western Europe and East Asia have implemented advanced sediment-inclusive monitoring frameworks, as exemplified by initiatives such as the EU’s MSFD and Korea’s Han River Basin Management Plan [5,89,125]. In contrast, many regions in South America, Africa, and Southeast Asia lack sufficient technical capacity, institutional support, and financial resources to implement comprehensive sediment monitoring and governance [5,129]. These disparities hinder global synthesis and exacerbate ecological risks in under-resourced areas [129]. Addressing these gaps requires tailored policy instruments, capacity-building efforts, and equitable international collaboration to support robust global monitoring and adaptive governance [5,130].

### 7.4. Strategic Integration of Monitoring and Governance

Deficiencies in integrating socio-behavioral dimensions into environmental governance are equally significant. Public risk perceptions, policy acceptance, and behavioral responses to plastic regulations vary widely across contexts [16,131,132]. In many cases, compliance is driven more effectively by incentive structures than by punitive bans [133]. Moreover, interdisciplinary collaboration among political scientists, behavioral economists, and legal scholars remains limited. Stronger engagement from these disciplines is essential for the co-development of policy instruments—such as plastic taxes, circular economy incentives, and deposit–return systems—that align both with ecological evidence and with public values [23,129].

Supplementary Table S1 emphasizes that plastic burdens in sediment and air can vary by several orders of magnitude globally, reflecting local governance efficacy, waste management infrastructure, and public participation levels. Without integrating these socio-political factors into monitoring and policy design, technical solutions alone will be insufficient to address the systemic nature of plastic pollution.

## 8. Transdisciplinary Strategies for Plastic Governance and Systemic Integration

This review reveals that plastics are both material pollutants and systemic disruptors embedded across environmental compartments. Physicochemical properties of plastics, particularly their durability, buoyancy, and reactivity, govern their transport through rivers, estuaries, and the atmosphere, as well as their long-term entrapment in sediments. Once retained, legacy plastics continue to influence ecosystem structure, contaminant dynamics, and sediment function [63,134,135,136].

Recent global data syntheses (Supplementary Table S1) have underscored sedimentary plastic burdens reaching over 27,000 PC/kg dw in certain riverine systems and exceeding 3000 PC/kg dw in multiple lake sediments. These alarming figures emphasize the urgency of adopting integrated monitoring and governance approaches capable of addressing both surface and subsurface compartments on a global scale.

Persistence of fragmented monitoring systems and methodological inconsistencies impedes our understanding of the ecological and policy consequences of plastic pollution. To overcome this, harmonized protocols and integrated monitoring strategies are urgently required. These should extend beyond surface observations and encompass sedimentary, biological, and airborne pathways. Global plastic governance must evolve from reactive cleanup to predictive management, grounded in comprehensive data integration and lifecycle-based modeling [4,5].

Effective governance must address plastic production, consumption, environmental leakage, and long-term ecological retention [5,129]. Concrete examples illustrate the feasibility of the integrated approaches [89]. For example, the European Union’s MSFD incorporates sediment monitoring as part of its comprehensive marine litter strategy, setting a precedent for sediment-inclusive surveillance [125]. The Thames River Programme in the United Kingdom exemplifies a multicompartment monitoring model by including riverbed sediments and biological compartments in long-term assessments [89]. Additionally, South Korea’s Han River Basin Management Plan has initiated pilot projects integrating microplastic tracking in both surface and sedimentary compartments, demonstrating a cross-scale governance approach that can be adapted regionally [129]. By analyzing and scaling these practical initiatives, policymakers can design adaptive frameworks that reflect both scientific evidence and local socio-political contexts [5].

Figure 7 presents a system-level framework that addresses this challenge, highlighting four intersecting domains—science, policy, ethics, and education—that must be integrated to manage plastics as systemic stressors. Governance in the Anthropocene demands not only enhanced international regulatory coordination but also upstream innovations in material design, expanded public engagement, and value-driven ethical considerations [5,23,137,138,139].

Incorporation of plastic flows into Earth system models, analogous to carbon and nitrogen cycles, is critical for forecasting ecological thresholds, simulating long-term legacy effects, and supporting evidence-based environmental policy decisions [66,140,141]. These models must integrate hydrological and biogeochemical feedback, regional governance structures, and human behavioral dynamics to ensure relevance across multiple policy scales [142,143,144].

Ultimately, plastics should be recognized not merely as pollutants but as persistent synthetic agents that fundamentally alter ecosystem structure and function. Thus, a systems-level approach spanning materials engineering, ecological science, and transnational policy is essential for addressing plastic flows as a defining challenge of the Anthropocene. Plastic governance must be embedded within Earth system governance frameworks, embracing principles of adaptive capacity, multilevel coordination, and ecological justice [145,146,147]. As shown in Figure 7, the effective management of plastic flows requires the coordinated integration of scientific metrics, ethical principles, regulatory instruments, and educational outreach, emphasizing that none of these domains can function in isolation. Such integrated governance not only enhances technical effectiveness but also reinforces public legitimacy, global comparability, and long-term systemic resilience. The above synthesis lays the foundation for concluding reflections, where we outline strategic imperatives for advancing global governance and comprehensive monitoring of plastic flows.

## 9. Conclusions

Plastic pollution, characterized by its non-cyclic flow and long-term environmental persistence, poses a complex and multifaceted challenge to both ecological and human health risk assessments. Unlike natural materials that circulate within closed biogeochemical loops, plastics accumulate in sediments and establish chronic exposure pathways in ecosystems. Recent global syntheses have documented sedimentary microplastic loads exceeding 27,000 PC/kg dw in certain river systems, highlighting the urgency to include sedimentary entrapment and legacy effects in comprehensive risk assessments.

Our review highlights the need to address sediment and subsurface compartments as critical sinks in policy and scientific frameworks. Current monitoring and policy approaches remain heavily biased toward surface water and visible macro debris, thereby underestimating the hidden but substantial risks posed by sedimentary and subsurface plastic burdens.

We propose a transdisciplinary governance framework that integrates scientific monitoring, Earth system modeling, policy reform, and public education to support a holistic and adaptive response. Strengthening sediment-focused monitoring and embedding plastics within water resource management and global risk assessment frameworks is crucial for accurately quantifying and mitigating risks.

Ultimately, addressing plastic pollution demands a fundamental shift from reactive cleanup strategies to proactive, systemic prevention rooted in sustainability science and long-term ecosystem resilience. By drawing lessons from regional programs, including Thames River sediment-inclusive assessments, South Korea’s Han River pilot studies, and the EU MSFD, policymakers can design more adaptable and context-specific frameworks that reflect both scientific evidence and local socio-political priorities. By integrating these insights into policy design and adaptive management practices, we can safeguard freshwater and marine ecosystems, mitigate chronic human health risks, and foster sustainable and resilient relationships with synthetic materials for future generations.

### Policy Recommendations

To translate these insights into concrete actions, we propose the following strategies:

Adopt sediment-inclusive monitoring frameworks inspired by successful regional programs, such as the EU MSFD, Thames River integrated monitoring, and South Korea’s Han River Basin initiatives.

Implement upstream reduction measures, including stricter controls on plastic production and use, improved waste management, and economic incentives for reusable and biodegradable alternatives. However, current waste management infrastructures are not yet fully adapted for the large-scale production, collection, and treatment of such alternatives, highlighting the urgent need for systemic redesign, technological innovation, and policy support.

Integrate cross-sectoral governance by combining environmental, public health, and socioeconomic policies to holistically address plastic flows

Enhance public awareness and education programs, emphasizing the ecological and health implications of microplastics, and promoting behavioral changes.

Incorporate plastic fluxes into Earth system models to forecast long-term legacy effects and guide evidence-based policy development.

These recommendations aim to foster proactive science-driven governance approaches that address plastics as a systemic stressor in the Anthropocene context.

## Data Availability

The data supporting the findings of this study are available from the corresponding author upon reasonable request.

## Supplementary Materials

The following supporting information can be downloaded at: https://www.mdpi.com/article/10.3390/nano15201546/s1, Table S1: Global concentrations of microplastics across environmental compartments and matrices (2015–2025); Table S2: Global concentrations of microplastics across environmental compartments and matrices (2015–2025); Table S3: Source data for the distribution of microplastic concentrations in regional and high-altitude freshwater sediments (Figure 5).
