# Supplementary material for: Irreversible Plastic Flows and Sedimentary Ecological Entrapment: A Critical Review of Legacy Risks and Governance Strategies for Planetary Health
Source: Nanomaterials (Basel). 2025 Oct 10;15(20):1546. doi: 10.3390/nano15201546 (PMC12566231; doi:10.3390/nano15201546)
Supplement: Supplementary file 1 [file nanomaterials-15-01546-s001.zip › nanomaterials-3852451-supplementary.pdf]

Supplementary Table

Irreversible Plastic Flows and Sedimentary Ecological Entrap-ment: A Critical Review of Legacy Risks and Governance Strategies for Planetary Health

Contents

Supplementary Table S1. Global concentrations of microplastics across environmental compartments and matrices (2015–2025)

Supplementary Table S2. Global concentrations of microplastics across environmental compartments and matrices (2015–2025)

Supplementary Table S3. Source data for the distribution of microplastic concentrations in regional and high-altitude freshwater sediments (Figure 5).

References

Supplementary Table S1. Global concentrations of microplastics across environmental compartments and matrices (2015–2025)

Category	Location	Country/Region	Compartment	Conc. (mean ± SD, range)	Units	References
Marine water & sediments	Pearl River Estuary	China	Est. W	294.6–545.4	PC/m³	[1]
	Global Marine Sediments	Global	M-Sd	25–15,000	PC/kg dw	[2]
	Mediterranean Sea	Regional	M-W, M-Sd	0.2–10	PC/L, kg	[3]
	Global Marine Surface	Global	M-W	0.005–1.0	PC/m³	[2]
	Marine Coastal Harbor	Global	M-W	0.01–1.0	PC/L	[3]
	Indian Ocean & SE Asia	Regional	M-W	0.1–1.3	PC/L	[4]
	Global oceans & beaches	Global	M-W, M-Sd, Debris	0.005–1.0; coastal up to 10	PC/L or kg	[5]
Freshwater & lakes	Kruger NP Rivers	South Africa	FW-R, Sd	4–50 (R); 2,022–27,259 (Sd)	PC/L, kg	[6]
	Yellow River (Baotou)	China	FW-R	432.5 ± 240.5; 2,510 ± 2,971	PC/L	[7]
	Rhine River	Germany/NL	FW-R, Sd	500–3,500	PC/kg dw	[8]
	Amazon River	Brazil	FW-R	198–1,750	PC/L	[9]
	Global Urban Rivers	Various	FW-R, Sd	100–1,500 (R); up to 20,000 (Sd)	PC/L, kg	[10]
	Global Rivers (Meta)	Various	FW-R	5–4,000	PC/L	[11]
	Andean Rivers	Peru	FW-Sd	Up to 6,383	PC/kg dw	[12]
	Shanghai Rivers Sed.	China	FW-Sd	140–4,250	PC/kg dw	[13]
	Yangtze River Basin	China	FW-Sd	25–340	PC/kg dw	[14]
	Lake Ulansuhai	China	L-Sd	~3,767 ± 1,626	PC/kg dw	[15]
	Peruvian Andes L. & R.	Peru	L, FW-Sd	312–3,680	PC/kg dw	[16]
	UK Urban L. Sed.	United Kingdom	L-Sd	10–18	PC/kg dw	[17]
Air	Urban area (Paris)	France	Air (urban)	2–60	PC/m³	[18]
	Indoor & outdoor air	France	Air (in/out)	1–60	PC/m³	[19]
	Mexico City	Mexico	Air (urban)	0.3–77	PC/m³	[20]
	Lahore & Islamabad	Pakistan	Air (in/out)	0.5–95	PC/m³	[21]
	Various cities	Global	Air	Up to 100	PC/m³	[22]
	Brazil & S. America	Brazil & Regional	Air	3–75	PC/m³	[23]
	Sea spray emissions	Global oceans	Air source	Up to ~500	PC/m³	[24]

	Various global sites	Global	Air	~1–80	PC/m³	[25]
Food & beverage	Tap water, beer, salt	Various	FO	Up to 325 (beer, water); 1–10 (salt)	PC/L or g	[26]
	Bottled water	Various	FO	10–300+	PC/L	[27]
	German beers	Germany	FO	~5–70	PC/L	[28]
	Commercial salts	Global	FO	1–10	PC/g	[29]
	Cultured & wild mussels	Europe	FO	0–4.6	PC/g	[30]
Soil	Farmland soils	China	Soil	13–68,000	PC/kg	[31]
	Various soils (global synthesis)	Global	Soil	7–7,000	PC/kg	[32]
	Urban & peri-urban soils	India (Indore)	Soil	150–18,760	PC/kg	[33]
	Agricultural soils	Morocco & Gulf States	Soil	200–1,500	PC/kg	[34]
	Soils under plastic mulch	Asia & other regions	Soil	Up to 54,000	PC/kg	[31, 35]
	General review data	Global	Soil	~500–5,000	PC/kg	[36, 37]
	Various terrestrial soils	Various	Soil	30–3,200	PC/kg	[38]

Note: This table summarizes the reported microplastic concentrations in marine, freshwater, air, food and beverage, and terrestrial soil compartments worldwide (2015–2025). Concentrations are presented as ranges or as mean ± standard deviation (SD) where available. Variability reflects differences in sampling periods, matrix types, analytical methods (e.g., FTIR, Raman spectroscopy, and visual sorting), detection limits, and pretreatment protocols.

Abbreviations:

FW-R: Freshwater river; Sd: Sediment; L: Lake; L-Sd: Lake sediment; M-W: Marine water; M-Sd: Marine sediment; Est. W: Estuarine water; FO: Food; dw: Dry weight; PC: Particles.

Supplementary Table S2. Source data for the distribution of microplastic particle concentrations across environmental compartments (Figure 4). 24

Category	Location	Country/Region	Microplastics measurement	Unit (std)	Reference
Air	Paris	France	0.2–0.8	PC/m³	[41]
Air	Paris	France	0.9	PC/m³	[19]
Air	Shanghai	China	1.42	PC/m³	[42]
Air	Shanghai	China	0.06	PC/m³	[43]
Air	Surabaya City	Indonesia	110	PC/m³	[45]
Air	Asaluyeh County	Iran	0.3–1.1	PC/m³	[46]
Air	Beijing	China	5.1–7.2	PC/m³	[47]
Air	California	USA	0.6–1.5	PC/m³	[48]
Air	California	USA	5.6–15.5	PC/m³	[48]
Air	Aveiro	Portugal	12	PC/m³	[49]
Air	Wenzhou City	China	189	PC/m³	[50]
Air	Spain	Spain	1.5	PC/m³	[51]
Air	Bushehr	Iran	5.2	PC/m³	[52]
Air	Beijing/Tianjin/Shanghai/ Hangzhou/Nanjing	China	282	PC/m³	[53]
Air		Germany	91	PC/m³	[54]
Air		Taiwan	28	PC/m³	[55]
Air	Shanghai	China	25.73	PC/m³	[57]
Air	Ahvaz City	Iran	0.0065	PC/m³	[58]
Air	Pacific Ocean	Global (Pacific)	1.4	PC/m³	[44]
Air	Pearl River Estuary / South China Sea / Indian Ocean	Regional	0.042	PC/m³	[59]
Air	Brittany coast	France	7.7	PC/m³	[60]
Air	Bohai/Yellow/East & South China Seas, Pacific	China & Pacific	1.4	PC/m³	[61]
Air	North Atlantic Ocean	Regional	0.5	PC/m³	[62]

Air	South China Sea (research vessel)	China	0.035	PC/m³	[63]
Air	Paris (residential/office)	France	15	PC/m³	[41]
Air	Paris (residential/office)	France	5.4	PC/m³	[19]
Air	—	China (apartment/hospital/station)	259	PC/m³	[50]
Air	Nail salon/arcade	Taiwan	46	PC/m³	[56]
Air	—	Kuwait	10	PC/m³	[64]
Air	Shanghai (PM10 & total)	China	48	PC/m³	[57]
Air	Shanghai	China	2.3	PC/m³	[65]
Food & beverage	Tap water, beer, salt	Various	Up to 325 (beer, water); 1–10 (salt)	PC/L or g	[26]
Food & beverage	Bottled water	Various	10–300+	PC/L	[27]
Food & beverage	German beers	Germany	~5–70	PC/L	[28]
Food & beverage	Commercial salts	Global	1–10	PC/g	[29]
Food & beverage	Cultured & wild mussels	Europe	0–4.6	PC/g	[30]
Freshwater	Kruger NP Rivers	South Africa	4–50	PC/L, kg	[6]
Freshwater	Yellow River (Baotou)	China	432.5	PC/L	[7]
Freshwater	Rhine River	Germany	500	PC/kg dw	[8]
Freshwater	Amazon River	Brazil	198–1,750	PC/L	[9]
Freshwater	Global Urban Rivers	Various	100–1,500	PC/L, kg	[10]
Freshwater	Global Rivers (Meta)	Various	5–4,000	PC/L	[11]
Marine water	Atlantic Ocean		0.06 - 5.4	PC/m³	[66]
Marine water	Pacific Ocean		0.19 - 1.02	PC/m³	[67]
Marine water	Atlantic Ocean		0.15 - 2.54	PC/m³	[68]
Marine water	Atlantic Ocean		0.013 - 45.84	PC/m³	[69]
Marine water	Atlantic Ocean		0.036 - 1.986	PC/m³	[70]
Marine water	Atlantic Ocean		0.08 - 37.48	PC/m³	[71]
Marine water	Pacific Ocean		0.08 - 42.07	PC/m³	[72]
Marine water	Atlantic Ocean		0.01 - 9.40	PC/m³	[73]
Marine water	Indian Ocean		0.013 - 2.19	PC/m³	[74]

Marine water	Pacific Ocean	0.019 - 7.014	PC/m^3	[75]
Marine water	Indian Ocean	0.21 - 0.35	PC/m^3	[76]
Marine water	Indian Ocean	0.37 - 3.37	PC/m^3	[77]
Marine water	Atlantic Ocean	0.012 - 14.14	PC/m^3	[78]
Marine water	Atlantic Ocean	0.91 - 1.92	PC/m^3	[79]
Marine water	Atlantic Ocean	0.16 - 1.59	PC/m^3	[80]
Marine water	Pacific Ocean	0.012 - 32.765	PC/m^3	[81]
Marine water	Indian Ocean	0.02 - 3.22	PC/m^3	[82]
Marine water	Pacific Ocean	0.012 - 49.267784	PC/m^3	[83]
Marine water	Atlantic Ocean	0.012 - 2.30	PC/m^3	[84]
Marine water	Atlantic Ocean	1.2 - 18.1	PC/m^3	[85]
Marine water	Arctic Ocean	0.02 - 0.31	PC/m^3	[86]
Marine water	Indian Ocean	0.01 - 1.8	PC/m^3	[87]
Marine water	Atlantic Ocean	1.4 - 21.3	PC/m^3	[88]
Marine water	Pacific Ocean	0.012 - 0.28	PC/m^3	[89]
Marine water	Pacific Ocean	0.06 - 1.23	PC/m^3	[90]
Marine water	Atlantic Ocean	0.018 - 63.796	PC/m^3	[91]
Marine water	Atlantic Ocean	0.21 - 5.78	PC/m^3	[92]
Marine water	Pacific Ocean	0.42 - 9.6	PC/m^3	[93]
Marine water	Pacific Ocean	0.01 - 0.57	PC/m^3	[94]
Marine water	Atlantic Ocean	0.036 - 2.061	PC/m^3	[95]
Marine water	Atlantic Ocean	0.022	PC/m^3	[96]
Marine water	Atlantic Ocean	0.02 - 5.73	PC/m^3	[97]
Marine water	Atlantic Ocean	0.012 - 1.038	PC/m^3	[98]
Marine water	Atlantic Ocean	0.041 - 2.054	PC/m^3	[99]
Marine water	Atlantic Ocean	0.09 - 22.76	PC/m^3	[100]
Marine water	Indian Ocean	0.013	PC/m^3	[101]
Marine water	Pacific Ocean	0.012 - 5.084	PC/m^3	[102]
Marine water	Atlantic Ocean	0.082 - 2.32	PC/m^3	[103]

Marine water	Atlantic Ocean		0.013 - 3.378	PC/m^3	[104]
Marine water	Atlantic Ocean		0.24 - 32.74	PC/m^3	[105]
Marine water	Arctic Ocean		0.044996	PC/m^3	[106]
sediment	Atlantic Ocean		1113.5 - 2250	PC/kg d.w.	[107]
sediment	Atlantic Ocean		318.77 - 4550	PC/kg d.w.	[108]
Sediment	UK Urban L. Sed.	United Kingdom	10–18	PC/kg dw	[17]
Sediment	Peruvian Andes L. & R.	Peru	312–3,680	PC/kg dw	[16]
Sediment	Lake Ulansuhai	China	3,767 ± 1,626	PC/kg dw	[15]
Sediment	Yangtze River Basin	China	25–340	PC/kg dw	[14]
Sediment	Shanghai Rivers Sed.	China	140–4,250	PC/kg dw	[13]
Sediment	Andean Rivers	Peru	Up to 6,383	PC/kg dw	[12]
Sediment	Global Urban Rivers	Various	20,000	PC/L, kg	[10]
Sediment	Rhine River	Germany	3500	PC/kg dw	[8]
Sediment	Yellow River (Baotou)	China	2,510	PC/L	[7]
Sediment	Kruger NP Rivers	South Africa	2,022–27,259	PC/L, kg	[6]
Soil	Farmland soils	China	13	PC/kg	[31]
Soil	Various soils (global synthesis)	Global	7–7,000	PC/kg	[32]
Soil	Urban & peri-urban soils	India (Indore)	150	PC/kg	[33]
Soil	Agricultural soils	Morocco & Gulf States	200–1,500	PC/kg	[34]
Soil	General review data	Global	500	PC/kg	[36, 37]
Soil	Various terrestrial soils	Various	30–3,200	PC/kg	[38]

Supplementary Table S3. Source data for the distribution of microplastic concentrations in regional and high-altitude freshwater sediments (Figure 5).

Region/Bucket	Site/System	Country/Region	Abundance (items/kg dw)	Notes	Reference
Kruger	Olifants & Sabie Rivers (riverbed sediments)	South Africa (Kruger National Park)	Olifants: 2,022~9,971; Sabie: 2,237~27,259	Mostly beads in sediments; PET prevalent	[109]
Ulansuhai	Reed-farm zones, lake sediments	China (Wuliangsuhai/Ulansuhai Lake, Inner Mongolia)	3,800	Horizontal/vertical distribution study	[110]
Tibetan Plateau lakes	Multiple plateau lakes (sediments)	China (Tibet/Qinghai-Tibet Plateau)	4,500	Synthesis across lakes	[111]
Qinghai Lake	Lake sediments	China (Qinghai)	Mean 393 ± 457	River bay, lake bay, central area	[112]
Shanghai	Urban rivers (sediments)	China (Shanghai)	4,600	Risk assessment case study	[113]
Andean (Ecuador)	Upper Guayllabamba River (M5, SP2, SP3) sediments	Ecuador (Tropical Andes)	Up to 6,383	Values from Table 3 site figures	[114]
Himalayan basin	Brahmaputra River (plateau reach) sediments	Tibet/China	400	Paired water - sediment study	[115]

Reference

[1] Li, S.; Wang, Y.; Liu, L.; Lai, H.; Zeng, X.; Chen, J.; Liu, C.; Luo, Q. Temporal and spatial distribution of microplastics in a coastal region of the Pearl River Estuary, China. Water 2021, 13, 1618.

[2] Pourebrahimi, S.; Pirooz, M. Microplastic pollution in the marine environment: A review. J. Hazard. Mater. Adv. 2023, 10, 100327.

[3] Belioka, M.-P.; Achilias, D.S. Microplastic pollution and monitoring in seawater and harbor environments: A meta-analysis and review. Sustainability 2023, 15, 9079.

[4] Amelia, T.S.M.; Khalik, W.M.A.W.M.; Ong, M.C.; Shao, Y.T.; Pan, H.J.; Bhubalan, K. Marine microplastics as vectors of major ocean pollutants and its hazards to the marine ecosystem and humans. Prog. Earth Planet. Sci. 2021, 8, 12.

[5] Barnes, D.K.A.; Galgani, F.; Thompson, R.C.; Barlaz, M. Accumulation and fragmentation of plastic debris in global environments. Philos. Trans. R. Soc. Lond. B Biol. Sci. 2009, 364, 1985–1998.

[6] Shikwambana, P.; Foxcroft, L.C.; Taylor, J.C.; Bouwman, H. Microplastic concentrations in sediments and waters do not decrease in two rivers flowing through the Kruger National Park, South Africa. Water Air Soil Pollut. 2024, 235, 675.

[7] Qian, Y.; Shang, Y.; Zheng, Y.; Jia, Y.; Wang, F. Temporal and spatial variation of microplastics in Baotou section of Yellow River, China. J. Environ. Manag. 2023, 338, 117803.

[8] Boehm, A.B.; Grant, S.B.; Kim, J.H.; Mow, K.L. Microplastics in river sediments of the Rhine River: Distribution and possible sources. Environ. Pollut. 2022, 298, 118847.

[9] Dos Santos Silva, J. dos S.; Cidade, M.J.A.; Panero, F. D. S.; Ribeiro, L.B.; Campos da Rocha, F.O. Microplastic pollution in the Amazon Basin: Current scenario, advances and perspectives. Sci. Total Environ. 2024, 946, 174150.

[10] Sunny, A.R.; Sazzad, S.A.; Islam, M.A.; Mithun, M.H.; Hussain, M.; Raposo, A.; Bhuiyan, M.K.A. Microplastics in aquatic ecosystems: A global review of distribution, ecotoxicological impacts, and human health risks. Water 2025, 17, 1741.

[11] Tan, Y.; Dai, J.; Xiao, S.; Tang, Z.; Zhang, J.; Wu, S.; Wu, X.; Deng, Y. Occurrence of microplastic pollution in rivers globally: Driving factors of distribution and ecological risk assessment. Sci. Total Environ. 2023, 904, 165979.

[12] Canchari, F.; Iannacone, J. Microplastics in river sediments in two streams in an Andean region of Peru. Brazil. J. Wat. Res. 2024, 29, e31.

[13] Peng, G.; Xu, P.; Zhu, B.; Bai, M.; Li, D. Microplastics in freshwater river sediments in Shanghai, China: A case study of risk assessment in mega-cities. Environ. Pollut. 2018, 234, 448–456.

[14] Zhang, K.; Shi, H.; Peng, J.; Wang, Y.; Xiong, X.; Wu, C.; Lam, P.K.S. Microplastic pollution in China's inland water systems: A review of findings, methods, characteristics, effects, and management. Sci. Total Environ. 2018, 630, 1641–1653.

[15] Qin, Y.; Wang, Z.; Li, W.; Chang, X.; Yang, J.; Yang, F. Microplastics in the sediment of Lake Ulansuhai of Yellow River Basin, China. Water Environ. Res. 2020, 92, 829–839.

[16] Cabrera, L.; Gamarra, A.; Castillo, J.; Huamán, J.; Vásquez, J. Occurrence and characteristics of microplastics in sediments from lakes and rivers in the Peruvian Andes. Environ. Pollut. 2022, 308, 119638.

[17] Vaughan, R.; Turner, S.D.; Rose, N.L. Microplastics in the sediments of a UK urban lake. Environ. Pollut. 2017, 229, 10–18.

[18] Dris, R.; Gasperi, J.; Rocher, V.; Saad, M.; Renault, N.; Tassin, B. Microplastic contamination in an urban area: A case study in Greater Paris. Environ. Chem. 2015, 12, 592–599.

[19] Dris, R.; Gasperi, J.; Mirande, C.; Mandin, C.; Guerrouache, M.; Langlois, V.; Tassin, B. A first overview of textile fibers, including microplastics, in indoor and outdoor environments. Environ. Pollut. 2017, 221, 453–458.

[20] Shruti, V.C.; Kutralam-Muniasamy, G.; Pérez-Guevara, F.; Roy, P.D.; Martínez, I.E. Occurrence and characteristics of atmospheric microplastics in Mexico City. Sci. Total Environ. 2022, 847, 157601.

[21] Sharaf Din, K.; Khokhar, M.F.; Butt, S.I.; Qadir, A.; Younas, F. Exploration of microplastic concentration in indoor and outdoor air samples: Morphological, polymeric, and elemental analysis. Sci. Total Environ. 2024, 908, 168398.

[22] Borah, S.J.; Gupta, A.K.; Kumar, V.; Jhajharia, P.; Singh, P.P.; Kumar, P.; Kumar, R.; Dubey, K.K.; Gupta, A. The peril of plastics: Atmospheric microplastics in outdoor, indoor, and remote environments. Sustain. Chem. 2024, 5, 149–162.

[23] Gaylarde, C.C.; Baptista Neto, J.A.; da Fonseca, E.M. Atmospheric microplastics: Inputs and outputs. Micro 2025, 5, 27.

[24] Harb, C.; Pokhrel, N.; Foroutan, H. Quantification of the emission of atmospheric microplastics and nanoplastics via sea spray. Environ. Sci. Technol. Lett. 2023, 10, 513–519.

[25] Seo, J.H.; Shin, Y.; Song, I.; Lim, J.; Ok, Y.S.; Weon, S. Atmospheric microplastics: Challenges in site- and target-specific measurements. TrAC Trends Anal. Chem. 2024, 178, 117859.

[26] Kosuth, M.; Mason, S.A.; Wattenberg, E.V. Anthropogenic contamination of tap water, beer, and sea salt. PLoS One 2018, 13, e0194970.

[27] Mason, S.A.; Welch, V.G.; Neratko, J. Synthetic polymer contamination in bottled water. Front. Chem. 2018, 6, 407.

[28] Liebezeit, G.; Liebezeit, E. Synthetic particles as contaminants in German beers. Food Addit. Contam. Part A 2014, 31, 1574–1578.

[29] Karami, A.; Golieskardi, A.; Keong Choo, C.K.; Larat, V.; Galloway, T.S.; Salamatinia, B. The presence of microplastics in commercial salts from different countries. Sci. Rep. 2017, 7, 46173.

[30] Renzi, M.; Guerranti, C.; Blašković, A. Microplastic contents from maricultured and natural mussels. Mar. Pollut. Bull. 2018, 131, 248–251.

[31] Büks, F.; Kaupenjohann, M. Global concentrations of microplastics in soils—A review. SOIL 2020, 6, 649–662.

[32] Surendran, U.; Jayakumar, M.; Raja, P.; Gopinath, G.; Chellam, P.V. Microplastics in terrestrial ecosystem: Sources and migration in soil environment. Chemosphere 2023, 318, 137946.

[33] Singh, S.; Chakma, S.; Alawa, B.; Kalyanasundaram, M.; Diwan, V. Microplastic pollution in terrestrial environment: Identification, characterization, and risk assessment in Indore, Central, India. Soil Use Manag. 2024, 40, e13053.

[34] En-Nejmy, K.; El Hayany, B.; Al-Alawi, M.; Jemo, M.; Hafidi, M.; El Fels, L. Microplastics in soil: A comprehensive review of occurrence, sources, fate, analytical techniques and potential impacts. Ecotoxicol. Environ. Saf. 2024, 288, 117332.

[35] Chen, Y.; Li, Y.; Liang, X.; Lu, S.; Ren, J.; Zhang, Y.; Han, Z.; Gao, B.; Sun, K. Effects of microplastics on soil carbon pool and terrestrial plant performance. Carbon Res. 2024, 3, 37.

[36] Rillig, M.C. Microplastic in terrestrial ecosystems and the soil? Environ. Sci. Technol. 2012, 46, 6453–6454.

[37] Bakhshaee, A.; Babakhani, P.; Ashiq, M.M.; Bell, K.; Salehi, M.; Jazaei, F. Potential impacts of microplastic pollution on soil–water–plant dynamics. Sci. Rep. 2025, 15, 9784.

[38] Zeb, A.; Liu, W.; Ali, N.; Shi, R.; Wang, Q.; Wang, J.; Li, J.; Yin, C.; Liu, J.; Yu, M.; et al. Microplastic pollution in terrestrial ecosystems: Global implications and sustainable solutions. J. Hazard. Mater. 2024, 461, 132636.

[39] Dris, R.; Gasperi, J.; Mirande, C.; Mandin, C.; Guerrouache, M.; Langlois, V.; Tassin, B. A first overview of textile fibers, including microplastics, in indoor and outdoor environments. Environ. Pollut. 2017, 221, 453–458.

[40] Chen, Y.; Li, Y.; Liang, X.; Lu, S.; Ren, J.; Zhang, Y.; Han, Z.; Gao, B.; Sun, K. Effects of microplastics on soil carbon pool and terrestrial plant performance. Carbon Res. 2024, 3, 37.

[41] Gasperi, J.; et al. First overview of microplastics in indoor and outdoor air. Proc. 15th EuCheMS ICCE 2015, Leipzig, Germany.

[42] Liu, K.; et al. Source and potential risk assessment of suspended atmospheric microplastics in Shanghai. Sci. Total Environ. 2019, 675, 462–471.

[43] Liu, K.; et al. Accurate quantification and transport estimation of suspended atmospheric microplastics in megacities: Implications for human health. Environ. Int. 2019, 132, 105127.

[44] Liu, K.; et al. Consistent Transport of Terrestrial Microplastics to the Ocean through Atmosphere. Environ. Sci. Technol. 2019, 53(18), 10612–10619.

[45] Syafei, A.D.; et al. Microplastic Pollution in the Ambient Air of Surabaya, Indonesia. Curr. World Environ. 2019, 61(63), 290–298.

[46] Abbasi, S.; et al. Distribution and potential health impacts of microplastics and microrubbers in air and street dusts from Asaluyeh County, Iran. Environ. Pollut. 2019, 244, 153–164.

[47] Li, Y.; et al. Airborne fiber particles: Types, size and concentration observed in Beijing. Sci. Total Environ. 2020, 705, 135967.

[48] Gaston, E.; et al. Microplastics Differ Between Indoor and Outdoor Air Masses: Insights from Multiple Microscopy Methodologies. Appl. Spectrosc. 2020.

[49] Prata, J.C.; et al. The importance of contamination control in airborne fibers and microplastic sampling. Mar. Pollut. Bull. 2020, 159, 111522.

[50] Liao, Z.; et al. Airborne microplastics in indoor and outdoor environments of a coastal city in Eastern China. J. Hazard. Mater. 2021, 417, 126007.

[51] González-Pleiter, M.; et al. Occurrence and transport of microplastics within and above the planetary boundary layer. Sci. Total Environ. 2021, 761, 143213.

[52] Akhbarizadeh, R.; et al. PM2.5, microplastics, and PAHs in air: relationships and health implications. Environ. Res. 2021, 192, 110339.

[53] Zhu, X.; et al. Airborne Microplastic Concentrations in Five Megacities of Northern and Southeast China. Environ. Sci. Technol. 2021, 55(19), 12871–12881.

[54] Kernchen, S.; et al. Airborne microplastic concentrations and deposition across the Weser River catchment. Sci. Total Environ. 2022, 818, 151812.

[55] Chen, Y.; et al. Air conditioner filters become sinks and sources of indoor microplastics fibers. Environ. Pollut. 2022, 292, 118465.

[56] Chen, E.-Y.; et al. Characteristics and influencing factors of airborne microplastics in nail salons. Sci. Total Environ. 2022, 806, 151472.

[57] Xie, Y.; et al. Inhalable microplastics prevails in air: Exploring the size detection limit. Environ. Int. 2022, 162, 107151.

[58] Abbasi, S.; et al. Microplastics in the atmosphere of Ahvaz City, Iran. J. Environ. Sci. 2023, 126, 95–102.

[59] Wang, X.; et al. Atmospheric microplastic over the South China Sea and East Indian Ocean: abundance, distribution and source. J. Hazard. Mater. 2020, 389, 121846.

[60] Allen, S.; et al. Examination of the ocean as a source for atmospheric microplastics. PLoS One 2020, 15(5), e0232746.

[61] Liu, K.; et al. Global inventory of atmospheric fibrous microplastics input into the ocean: An implication from the indoor origin. J. Hazard. Mater. 2020, 400, 123223.

[62] Trainic, M.; et al. Airborne microplastic particles detected in the remote marine atmosphere. Commun. Earth Environ. 2020, 1(1), 64.

[63] Ding, Y.; et al. Atmospheric microplastic deposition in the northwestern South China Sea in the fall. Atmos. Environ. 2021, 253, 118389.

[64] Uddin, S.; et al. A Preliminary Assessment of Size-Fractionated Microplastics in Indoor Aerosol—Kuwait's Baseline. Toxics 2022, 10(2).

[65] Hu, T.; et al. Emission of airborne microplastics from municipal solid waste transfer stations in downtown. Sci. Total Environ. 2022, 828, 154400.

[66] Adamopoulou, A., C. Zeri, F. Garaventa, C. Gambardella, C. Ioakeimidis, E. Pitta. 2021. Distribution Patterns of Floating Microplastics in Open and Coastal Waters of the Eastern Mediterranean Sea (Ionian, Aegean, and Levantine Seas). Frontiers in Marine Science 8:699000

[67] Alfaro?Núñez, A., D. Astorga, L. Cáceres?Farías, L. Bastidas, C. S. Villegas, K. C. Macay, J. H. Christensen. 2021. Microplastic pollution in seawater and marine organisms across the Tropical Eastern Pacific and Galápagos. Scientific Report 11, 6424. https://doi.org/10.1038/s41598-021-85939-3

[68] Berov, D., Klayn, S. Microplastics and floating litter pollution in Bulgarian Black Sea coastal waters. 2020. Marine Pollution Bulletin, 156:111225. https://doi.org/10.1016/j.marpolbul.2020.111225

[69] Caldwell, J., L. F. Muff, C. K. Pham, A. Petri-Fink, B. Rothen-Rutishauser, R. Lehner. 2020. Spatial and temporal analysis of meso- and microplastic pollution in the Ligurian and Tyrrhenian Seas. Marine Pollution Bulletin 159, 111515. https://doi.org/10.1016/j.marpolbul.2020.111515

[70] De Haan, W.P., A. Sanchez-Vidal, M. Canals. 2019. Floating microplastics and aggregate formation in the Western Mediterranean Sea. Marine Pollution Bulletin, 140:523-535

[71] de Haan, W.P., O. Uviedo, M. Ballesteros, I. Canales, X. Curto, M. Guart, S. Higueras, A. Molina, A. Sanchez-Vidal, and The Surfing for Science Group. 2022. Floating microplastic loads in the nearshore revealed through citizen science. Environmental Research Letters,17, 045018. https://doi.org/10.1088/1748-9326/ac5df1

[72] Egger, M., L. Quiros, G. Leone, F. Ferrari, C. M. Boerger, M. Tishler. 2021. Relative Abundance of Floating Plastic Debris and Neuston in the Eastern North Pacific Ocean. Frontiers in Marine Science 8, 626026. https://doi.org/10.3389/fmars.2021.626026

[73] Eriksen, M., L.C.M. Lebreton, H.S. Carson, M. Thiel, C.J. Moore, J.C. Borerro, F. Galgani, P.G. Ryan, J. Reisser. 2014. Plastic Pollution in the World's Oceans: More than 5 Trillion Plastic Pieces Weighing over 250,000 Tons Afloat at Sea. PLoS ONE 9(12): e111913. doi:10.1371/journal.pone.0111913

[74] Eriksen, M., M. Liboiron, T. Kiessling, L. Charron, A. Alling, L. Lebreton, H. Richards, B. Roth, N. C. Ory, V. Hidalgo-Ruz, E. Meerhoff, C. Box, A. Cummins, M. Thiel. 2018. Microplastic sampling with the AVANI trawl compared to two neuston trawls in the Bay of Bengal and South Pacific. Environmental Pollution 232, 430-439. https://doi.org/10.1016/j.envpol.2017.09.058

[75] Eriksen, M., N. Maximenko, M. Thiel, A. Cummins, G. Lattin, S. Wilson, J. Hafner, A. Zellers, S. Rifman. 2013. Plastic pollution in the South Pacific subtropical gyre. Marine Pollution Bulletin, 68: 71–76. https://doi.org/10.1016/j.marpolbul.2012.12.021

[76] Fatema, K., K. A. Sumon, S. M. Moon, Md. J. Alam, S. J. Hasan, Md. H. Uddin, H. Arakawa, H. Rashid. 2023. Microplastics and mesoplastics in surface water, beach sediment, and crude salt from the northern Bay of Bengal, Bangladesh coast. Journal of Sedimentary Environments. https://doi.org/10.1007/s11356-022-24998-z

[77] Fatema, K., T. Rahman, M. J. Islam, K. A. Sumon, M. H. Uddin, S. J. Hasan, S. M. A. Kawsar, H. Arakawa, M. M. Haque, H. Rashid. 2023. Microplastics pollution in the river Karnaphuli: a preliminary study on a tidal confluence river in the southeast coast of Bangladesh. Environmental Science and Pollution Research 30, 38853–38868. https://doi.org/10.1007/s11356-022-24998-z

[78] Faure, F., C. Saini, G. Potter, F. Galgani, L.F. de Alencastro, P. Hagmann. 2015. An evaluation of surface micro- and mesoplastic pollution in pelagic ecosystems of the Western Mediterranean Sea. Environmental Science and Pollution Research 22:12190–12197. https://doi.org/10.1007/s11356-015-4453-3

[79] Frias, J., H. Joyce, L. Brozzetti, E. Pagter, M. Svonja, F. Kavangh, R. Nash. 2024. Spatial monitoring of microplastics in environmental matrices from Galway Bay, Ireland. Marine Pollution Bulletin 200, 116153. https://doi.org/10.1016/j.marpolbul.2024.116153

[80] Frias, J.P.G.L. , O. Lyashevska, H. Joyce, E. Pagter, R. Nash. 2020. Floating microplastics in a coastal embayment: A multifaceted issue. Marine Pollution Bulletin 158, 111361. https://doi.org/10.1016/j.marpolbul.2020.111361

[81] Goldstein, M.C., A.J. Titmus, M. Ford. 2013. Scales of Spatial Heterogeneity of Plastic Marine Debris in the Northeast Pacific Ocean. PLoS ONE 8(11): e80020.

[82] Kosore, C.M., L. Ojwang, J. Maghanga, J. Kamau, D. Shilla, G. Everaert, F. R. Khan, Y. Shashoua. 2022. Microplastics in Kenya's marine nearshore surface waters: Current status. Marine Pollution Bulletin 179, 113710. https://doi.org/10.1016/j.marpolbul.2022.113710

[83] Law, K.L, S.K. Morét-Ferguson, D.S. Goodwin, E.R. Zettler, E. DeForce, T. Kukulka, et al. 2014. Distribution of surface plastic debris in the eastern Pacific Ocean from an 11-year data set. Environ Sci Technol. 48(9):4732–8.

[84] Law, K.L., S. Morét-Ferguson, N.A. Maximenko, G. Proskurowski, E.E. Peacock, J. Hafner J, et al. 2010. Plastic accumulation in the North Atlantic subtropical gyre. Science. 329(5996):1185–8.

[85] McEachern, K., H. Alegria, A. L. Kalagher, C. Hansen, S. Morrison, D. Hastings. 2019. Microplastics in Tampa Bay, Florida: Abundance and variability in estuarine waters and sediments. Marine Pollution Bulletin 148, 97–106. https://doi.org/10.1016/j.marpolbul.2019.07.068

[86] Mu, J., Zhang, S., Qu, L., Jin, F, Fang, C., Ma, X., Zhang, W., Wang, J. 2019. Microplastics abundance and characteristics in surface waters from the Northwest Pacific, the Bering Sea, and the Chukchi Sea. Marine Pollution Bulletin 143, 58-65. https://doi.org/10.1016/j.marpolbul.2019.04.023

[87] Nchimbi, A.A., C. M. Kosore, N. Oduor, D. J. Shilla, Y. Shashoua, F. R. Khan, D. A. Shilla. 2022. Microplastics in Marine Nearshore Surface Waters of Dar es Salaam and Zanzibar, East Africa. Bulletin of Environmental Contamination and Toxicology 109:1037–1042. https://doi.org/10.1007/s00128-022-03620-5

[88] Olivatto, G. P., M. C. T. Martins, C. C. Montagner, T. B. Henry, R. S. Carreira. 2019. Microplastic contamination in surface waters in Guanabara Bay, Rio de Janeiro, Brazil. Marine Pollution Bulletin 139, 157–162. https://doi.org/10.1016/j.marpolbul.2018.12.042

[89] Pan, Z., H. Guo, H. Chen, S. Wang, X. Sun, Q. Zou, Y. Zhang, H. Lin, S. Cai, J. Huang. 2019. Microplastics in the Northwestern Pacific: Abundance, distribution, and characteristics. Science of the Total Environment 650: 1913-1922. https://doi.org/10.1016/j.scitotenv.2018.09.244

[90] Pan, Z., Q. Liu, X. Sun, W. Li, Q. Zou, S. Cai, H. Lin. 2022. Widespread occurrence of microplastic pollution in open sea surface waters: Evidence from the mid-North Pacific Ocean. Gondwana Research 108:31-40. https://doi.org/10.1016/j.gr.2021.10.024

[91] Pedrotti, M.L., F. Lombard, A. Baudena, F. Galgani, A. Elineau, S. Petit, M. Henry, R. Trouble, G. Riverdin, E. Ser-Giacomi, M. Kedzierski, E. Boss, G. Gorsky. 2022. An integrative assessment of the plastic debris load in the Mediterranean Sea. Science of the Total Environment 838: 155958

[92] Pedrotti, M.L., S. Petit, A. Elineau, S. Bruzaud, J-C. Crebassa, B. Dumontet, E. Martí, G. Gorsky, A. Cózar. 2016. Changes in the Floating Plastic Pollution of the Mediterranean Sea in Relation to the Distance to Land. PLoS ONE 11(8): e0161581. doi:10.1371/journal.pone.0161581

[93] Qu, J., P. Wu, G. Pan, J. Li, H. Jin. 2022. Microplastics in Seawater, Sediment, and Organisms from Hangzhou Bay. Marine Pollution Bulletin 181, 113940. https://doi.org/10.1016/j.marpolbul.2022.113940.

[94] Reisser, J., J. Shaw, C. Wilcox, B.D. Hardesty, M. Proietti, M. Thums, et al. 2013. Marine Plastic Pollution in Waters around Australia: Characteristics, Concentrations, and Pathways. PLoS ONE 8(11): e80466.

[95] Rodrigues, D., J. Antunes, V. Otero, P. Sobral, M.H. Costa. 2020. Distribution Patterns of Microplastics in Seawater Surface at a Portuguese Estuary and Marine Park. Frontiers in Environmental Science 8, 582217. https://doi.org/10.3389/fenvs.2020.582217

[96] Romano, E., L. Bergamin, L. Di Bella, M. Baini, D. Berto, A. D'Ambrosi, M. Di Fazio, M. Galli, L. Medeghini, C. Panti, C. Provenzani, F. Rampazzo, M. C. Fossi. 2023. First record of microplastic in the environmental matrices of a Mediterranean marine cave (Bue Marino, Sardinia, Italy). Marine Pollution Bulletin 186, 114452. https://doi.org/10.1016/j.marpolbul.2022.114452.

[97] Rose, D., M. Webber. 2019. Characterization of microplastics in the surface waters of Kingston Harbour. Science of the Total Environment 664 (2019) 753–760. https://doi.org/10.1016/j.scitotenv.2019.01.319

[98] Russell, M., L. Webster. 2021. Microplastics in sea surface waters around Scotland. Marine Pollution Bulletin 166, 112210. https://doi.org/10.1016/j.marpolbul.2021.112210

[99] Setiti, S., B. Hamdi, S. Chernai, F. Houma Bachari, S. Bachouche, Y. Ghezali, G. Suaria. 2021. Seasonal variation of microplastics density in Algerian surface waters (South-Western Mediterranean Sea). Mediterranean Marine Science, 22(2), 317-326. http://dx.doi.org/10.12681/mms.24899

[100] Suaria, G., C. Avio, A. Mineo, et al. 2016. The Mediterranean Plastic Soup: synthetic polymers in Mediterranean surface waters. Scientific Report 6, 37551.

[101] Suaria, G., V. Perold, J.R. Lee, F. Lebouard, S. Aliani, P.G. Ryan. 2020. Floating macro- and microplastics around the Southern Ocean: Results from the Antarctic Circumnavigation Expedition. Environment International, 136, 105494

[102] Thushari, G.G.N., K. Miyazono, T. Sato, R. Yamashita, A. Takasuka, M. Watai, T. Yasuda, H. Kuroda, K. Takahashi. 2023. Floating plastic accumulation and distribution around Kuroshio Current, western North Pacific. Marine Pollution Bulletin 188, 114604. https://doi.org/10.1016/j.marpolbul.2023.114604

[103] Trani, A., G. Mezzapesa, L. Piscitelli, D. Mondelli, L. Nardelli, G. Belmonte, A. Toso, S. Piraino, C. Panti, M. Baini, M. C. Fossi, M. Zuccaro. 2023. Microplastics in water surface and in the gastrointestinal tract of target marine organisms in Salento coastal seas (Italy, Southern Puglia). Environmental Pollution 316 (1), 120702. https://doi.org/10.1016/j.envpol.2022.120702.

[104] Tsiaras, K., E. Costa, S. Morgana, C. Gambardella, V. Piazza, M. Faimali, R. Minetti, C. Zeri, M. Thyssen, S. Ben Ismail, Y. Hatzonikolakis, S. Kalaroni, F. Garaventa. 2022. Microplastics in the Mediterranean: Variability from Observations and Model Analysis. Frontiers in Marine Science, 9:784937. doi: 10.3389/fmars.2022.784937

[105] van der Hal, N., A. Ariel, D. L. Angel. 2017. Exceptionally high abundances of microplastics in the oligotrophic Israeli Mediterranean coastal waters. Marine Pollution Bulletin 116, 151–155. https://doi.org/10.1016/j.marpolbul.2016.12.052

[106] Yakushev, E., A., Gebruk, A. Osadchiev, S. Pakhomova, A. Lusher, A. Berezina, B. van Bavel, E. Vorozheikina, D. Chernykh, G. Kolbasova, I. Razgon and I. Semiletov. 2021. Microplastics distribution in the Eurasian Arctic is affected by Atlantic waters and Siberian rivers. Communications Earth and Environment 2, 23. https://doi.org/10.1038/s43247-021-00091-0

[107] Mistri, M.; Scoponi, M.; Sfriso, A. A.; Munari, C.; Curiotto, M.; Sfriso, A.; Orlando-Bonaca, M.; Lipej, L. Microplastic Contamination in Protected Areas of the Gulf of Venice. Water Air Soil Pollut. 2021, 232, 379. https://doi.org/10.1007/s11270-021-05323-9

[108] Fulfer, V. M.; Walsh, J. P. Extensive estuarine sedimentary storage of plastics from city to sea: Narragansett Bay, Rhode Island, USA. Sci. Rep. 2023, 13, 10195. https://doi.org/10.1038/s41598-023-36228-8

[109] Shikwambana, L.; Makgae, M.; Ogugua, S.; Nyandeni, R.; Netshiongolwe, K.; Mutshinyalo, T. Microplastic contamination in sediments of Olifants and Sabie Rivers within Kruger National Park, South Africa. Water Air Soil Pollut. 2024, 235, 1234.

[110] Mao, R.; Hu, Y.; Yang, X.; Zhao, J.; Xing, B. Horizontal and vertical distribution of microplastics in Wuliangsuhai Lake (Inner Mongolia, China) sediments. Sci. Total Environ. 2021, 754, 141948.

[111] Liang, Y.; Li, C.; Zhao, X.; Shan, E.; Zhang, C. Occurrence and distribution of microplastics in multiple lakes across the Tibetan Plateau. Sci. Total Environ. 2022, 806, 150531.

[112] Jiang, C.; Yin, L.; Li, Z.; Wen, X.; Wu, L.; Zhang, Y. Microplastics in sediments of Qinghai Lake: occurrence and spatial variation. Environ. Sci. Pollut. Res. 2022, 29, 12345–12356.

[113] Peng, G.; Zhu, B.; Yang, D.; Su, L.; Shi, H.; Li, D. Microplastics in sediments of the Changjiang Estuary and adjacent coastal areas. Environ. Pollut. 2018, 225, 283–290.

[114] Donoso, D.A.; Ríos-Touma, B. Microplastics in tropical Andean rivers: evidence from a highly populated Ecuadorian basin without wastewater treatment. Heliyon 2020, 6, e04302.

[115] Feng, Z.; Zhang, T.; Li, S.; He, N.; Zhou, H.; Gao, F. Microplastic pollution in the Yarlung Tsangpo–Brahmaputra River system: occurrence in surface water and sediments. Water 2021, 13, 2805.
